# Identification of Relevant Attributes for Liver Cancer Therapies (IRALCT): a maximum-difference-scaling analysis

**DOI:** 10.1038/s41598-022-23097-w

**Published:** 2022-11-09

**Authors:** Bennet Hensen, Carolin Winkelmann, Frank K. Wacker, Bodo Vogt, Cornelia L. A. Dewald, Thomas Neumann

**Affiliations:** 1 Research Campus STIMULATE, Otto von Guericke University Magdeburg, Otto-Hahn-Straße 2, 39106 Magdeburg, Germany; 2 Chair in Empirical Economics, Otto von Guericke University Magdeburg, Universitätsplatz 2, 39106 Magdeburg, Germany; 3 Department of Diagnostic and Interventional Radiology, Hannover Medical School, Carl-Neuberg-Straße 1, 30625 Hannover, Germany; 4 Chair in Health Economics, Institute of Social Medicine and Health Systems Research, Otto-Von-Guericke-University Magdeburg, Leipziger Str. 44, 39120 Magdeburg, Germany; 5 University Department of Neurology, Otto von Guericke University Magdeburg, Leipziger Str. 44, 39120 Magdeburg, Germany; 6 Chair in Health Services Research, School of Life Sciences, University of Siegen, Am Eichenhang 50, 57076 Siegen, Germany

**Keywords:** Cancer therapy, Cancer

## Abstract

The Identification of Relevant Attributes for Liver Cancer Therapies (IRALCT) project is intended to provide new insights into the relevant utility attributes regarding therapy choices for malignant primary and secondary liver tumors from the perspective of those who are involved in the decision-making process. It addresses the potential value of taking patients’ expectations and preferences into account during the decision-making and, when possible, adapting therapies according to these preferences. Specifically, it is intended to identify the relevant clinical attributes that influence the patients’, medical laymen’s, and medical professionals’ decisions and compare the three groups’ preferences. We conducted maximum difference (MaxDiff) scaling among 261 participants (75 physicians, 97 patients with hepatic malignancies, and 89 medical laymen) to rank the importance of 14 attributes previously identified through a literature review. We evaluated the MaxDiff data using count analysis and hierarchical Bayes estimation (HB). Physicians, patients, and medical laymen assessed the same 7 attributes as the most important: probability (certainty) of a complete removal of the tumor, probability of reoccurrence of the disease, pathological evidence of tumor removal, possible complications during the medical intervention, welfare after the medical intervention, duration and intensity of the pain, and degree of difficulty of the medical intervention. The cumulative relative importance of these 7 attributes was 88.3%. Our results show that the physicians’, patients’, and medical laymen’s preferences were very similar and stable.

**Trial registration** DRKS-ID of the study: DRKS00013304, Date of Registration in DRKS: 2017/11/16.

## Introduction

The liver is a frequent site of metastatic disease for a broad variety of tumor entities, including gastrointestinal tract cancers (such as colorectal carcinoma) as well as breast and lung cancer, whereas hepatocellular carcinoma (HCC) is the predominant form of primary hepatic cancer^1,2^. Surgical resection can offer a curative treatment option^3^ for primary and secondary hepatic malignancies^4^. In addition, minimally invasive and locoregional therapies have proven to be safe and effective alternatives with low complication rates. Interdisciplinary tumor boards usually make the treatment decision for patients with malignant liver tumors. Although treatment guidelines provide a framework for various entities, stages, and treatment options (e.g., surgery versus ablation in early HCC^4^), choosing the treatment that best fits the individual patient can be challenging. Besides the variances in effectiveness and safety profiles, therapeutic methods can differ in other characteristics or attributes, such as intensity of the peri-procedural pain and the duration of hospitalization.

In the selection of the ideal therapeutic regimen, the patients’ preferences for certain attributes should be integrated into the decision-making process^5^. This emphasis on a more participatory role for patients is probably best known as shared decision-making^6^, which builds on the involvement of at least two partners (e.g., physician and patient). All parties involved take steps to participate in the treatment decision, share information about treatment options, and achieve consensus on a preferred treatment option^3,7^. A growing body of literature shows that shared decision-making can offer a variety of benefits, such as improved patient satisfaction, clinical outcomes, and disease management^5,7,8^. Still, exercising shared decision-making in clinical practice can be difficult. Even though the patient’s needs and wishes are taken into account in the discussion of treatment options in tumor boards, full shared decision-making in terms of a dialogue between a physician and a patient whose expertise is at the same level tends to be the exception rather than the rule.

In several studies, researchers have identified preferences in therapy choices across a broad variety of diseases^9–16^, including studies focused on distinct types of cancer^17–23^. Furthermore, in multiple clinical studies (e.g., phase 3 studies), researchers have examined various forms of treatment based on clinical attributes^24–26^. However, most of the available studies focus on either physicians’ or patients’ preferences but do not focus on both groups together. Therefore, besides a lack of knowledge on patients’ preferences in liver cancer therapy choices^27^, we do not know much about how physicians’ and patients’ preferences are related. Given the underlying topic of shared decision-making, we deem considering both perspectives (patients’ and physicians’) together important.

The main objective of our study is to address this gap in the literature by ascertaining which attributes influence patients’, physicians’, and medical laymen’s preferences when they choose liver cancer therapies. Specifically, we used conjoint analysis to synthesize findings to estimate utility functions and analyze potential differences among the groups.

In this regard, we designed the *Identification of Relevant Attributes for Liver Cancer Therapies* (IRALCT) study, which is a two-phase mixed-methods study aimed at providing new insights into the relevant clinical attributes for malignant hepatic tumor therapy decision-making from the perspective of those who are involved in the decision-making process. We ask whether it is worth taking the individual patient’s wishes and preferences into account and, if possible, aligning therapies and the decision-making process with these preferences. Ideally, our results should contribute to the design of a preference-based, shared decision-making process.

## Methods

### Study design

The IRALCT study is a two-phase mixed-methods study. In the first phase, we used the maximum difference scaling method (MaxDiff, a type of conjoint analysis) to determine which relevant clinical attributes can influence a treatment decision. In the second phase, we will evaluate therapies that have been adapted according to the results obtained in the first study phase. Because the second phase is ongoing, we did not include it in this article.

During the first study phase, we used MaxDiff scaling to identify patients’ and physicians’ preferences regarding certain relevant clinical attributes that influence the decision for hepatic-malignancy therapies. MaxDiff, also known as best‐worst scaling, is a standard method used to analyze participants’ preferences. Szeinbach et al.^28^ first applied it in health care, and McIntosh and Louviere^29^ formally introduced it into health care research. MaxDiff consists of choice tasks with a minimum of three options whereby the participant is asked to indicate the best and worst option in each choice task^30^. As MaxDiff is considered to represent a low cognitive burden on study participants^31^, it is an attractive and relatively easy method to investigate preferences over a wide range of health care topics^30^.

Prior to our computer-based study, we conducted a literature review to identify a preselection of clinical attributes relevant in the decision for selection of hepatic-malignancy therapies. We restricted the initial literature review to articles published between 2007 and 2017. We updated the literature review in 2021 to include recent publications and to determine whether the results derived from the review are still evident. We searched four databases, PubMed, Scopus, ScienceDirect, and CENTRAL (Cochrane Central Register of Controlled Trials), and used the Google Scholar search engine. To achieve a large number of relevant hits, we defined search terms and used multiple combinations with Boolean operators. We compiled search terms from five major categories: (1) names of (health related) conjoint analyses (e.g., “discrete choice experiment,” “choice experiment,” “choice based conjoint analysis,” “best worst scaling,” “maximum difference scaling,” “adaptive conjoint analysis,” “adaptive choice based conjoint analysis,” “conjoint analysis,” and “preference measurement”) because conjoint analyses are always connected to measuring attributes^32^, (2) cancer terminology (e.g., “carcinoma,” “tumour,” “oncology,” “metastatic,” “incurable,” “overall survival”), (3) general liver-cancer treatment preference terminology (e.g., “patients’ preferences,” “physicians’ preferences,” “treatment attributes,” “risk of side effects,” “costs,” “treatment time,” “comfortability”), (4) disease-specific therapies and terms (e.g., “transcatheter arterial chemoembolization” (TACE), “selective internal radiation therapy” (SIRT), “chemosaturation,” “ablation,” “efficacy,” “safety”), and (5) health-related quality of life assessment terminology (e.g., “Patient-Reported Outcome” (PRO), “Health-Related Quality of Life” (HrQoL), “Quality of Life” (QoL), “health status,” “health status indicator,” “functional status,” “subjective health status,” “health status assessment,” “EQ-5D,” “SF-36,” and “QLQ C-30”). Note that the search terms listed for each category represent an exemplary selection of a larger set of search terms.

Of the studies we found, we included only those that measure attributes and deal with malignant primary and secondary liver tumors. We supplemented this set of studies with studies found from other sources. After removing duplicates, two reviewers assessed the titles and abstracts of the remaining set of studies for relevance. Afterward, two reviewers retrieved and reviewed the full-text documents and determined whether we would include the papers in the final review based on the eligibility criteria (“Supplementary information”).

One key finding was that in most studies, the researchers surveyed either a group of physicians or a group of patients with questions or attributes adapted to the respective group. In studies focusing on patients, researchers have evaluated alternative treatment options from personal and social perspectives, whereas in studies focusing on physicians, researchers have often evaluated treatments’ efficiency in rather objective and measurable factors, which, furthermore, are specific to the type of tumor and available treatment methods. Physicians and patients could arrive at different results simply because of the type of question or framing of the facts. Therefore, based on the available studies, it is not possible to determine whether physicians’ and patients’ preferences are aligned. Furthermore, this fact makes it difficult to derive joint preferences and to identify attributes that are important for both groups involved in the shared decision-making process.

To overcome this problem, in the current study, we presented patients and physicians with the same evaluation criteria.

In the final review, we derived attribute categories that we considered relevant. For example, Li et al.^33^, Lo et al.^2^, and Puts et al.^34^ reported overall survival was the most important, followed by the risk of side effects and adverse events^2,34^. Other categories considered relevant in the studies are costs^35^, treatment time^36–38^, comfortability^39^, and administration^2,36–38,40^. Based on the categories we preselected this way, we determined 14 specific clinical attributes, presented in Table 1, being careful to ensure that patients, physicians, and medical laymen could evaluate them. In the Supplementary information we present these attributes in more detail.

**Table 1:** Decision-relevant attributes.

Attribute no.	Attribute
1	Probability (certainty) of a complete removal of the tumor
2	Probability of reoccurrence of the disease
3	Pathological evidence of tumor removal
4	Possible complications during the medical intervention
5	Welfare after the medical intervention
6	Duration and intensity of the pain
7	Degree of difficulty of the medical intervention
8	Frequency of follow-up care
9	Type of anesthesia
10	Duration of hospitalization
11	Length of anesthesia
12	Cost of therapy
13	Size of the scar
14	Profit of the hospital as a result of the treatment choice

To ensure that the groups answered the questions from their intended perspectives, we framed the participants accordingly in advance of the survey. We contacted the group of physicians with a personal letter in which we explicitly asked for their professional assessment. The patients were in an appropriate setting due to their stay in the hospital during the survey, but we also explicitly asked for their assessment from the patient’s point of view. In addition, we asked all respondents at the end whether they had answered the questions from a physician’s, patient’s, or medical laymen’s point of view.

**Figure 1:**
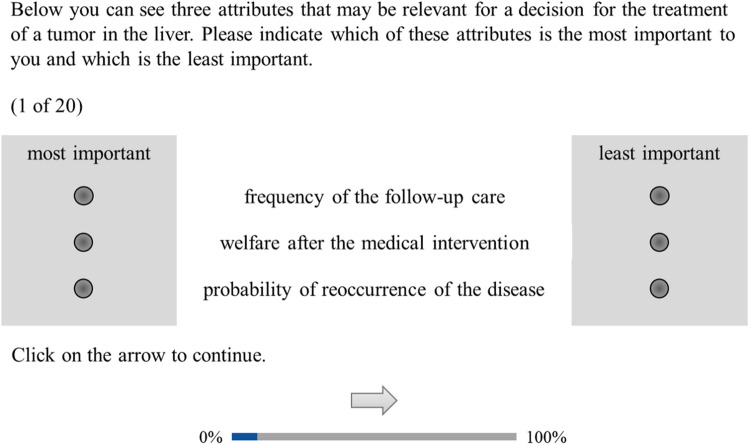
Screenshot of the MaxDiff (maximum-difference scale) choice task.

During the MaxDiff, we asked the participants to assess choice tasks. In each one, we asked the participants to evaluate three of these attributes and indicate which was (1) the most important and (2) the least important. We presented the participants with twenty choice tasks. Figure 1 presents an example of a choice task. We retrieved the participants’ demographic and clinical characteristics after completing the MaxDiff.

We conducted the study between November 2017 and September 2019 at Hannover Medical School, a tertiary referral hospital in Germany. The local ethics committee reviewed and approved all data management and outcome-related activities of this research project. Patients provided their written consent to participate in the study prior to any examination.

### Study size

We used Sawtooth Software to collect and evaluate the data. We calculated the sample size for the MaxDiff in accordance with the recommendations from Sawtooth. Because we (1) were interested in the individual preferences regarding the relevant clinical attributes within each group of participants (patients, physicians, and medical laymen) and (2) wanted to compare the obtained preference orders from the three groups, we had to calculate the required sample size for each group. In total, we included 14 items in the study. In each task, we displayed three items per set, and the total number of tasks per participant was 20. We defined these three criteria—(1) total number of tasks per participant, (2) selected number of items, and (3) displayed items per set—in advance. We calculated the item frequency per participant by dividing the displayed items per set by the product of selected number of items and total number of tasks per participant. According to Sawtooth Software, each attribute should be rated at least 500 times across all participants^41^. This resulted in a required sample size of 117 per group (see Table 2). For a pooled analysis across all groups, the optimal sample size was between 117 and 233.

**Table 2:** Sample-size calculation.

Total number of tasks per participant	20
Selected number of items	14
Displayed items per set	3
Item frequency per participant	4.3
Sample size	117

### Participants – inclusion and exclusion criteria

The study was focused on patients who were receiving or had received a minimally invasive tumor treatment or surgical treatment. Further inclusion criteria for patients included age (at least 18 years old) and ability to consent. The main exclusion criteria were age (< 18 years old) and a critical or unstable state of health. Furthermore, we excluded patients who had participated in another survey or study within the last 30 days. We conducted the interviews during the in-patient stay. Participants completed the computer-based survey using a tablet computer. An interviewer was available during the survey to answer questions and provide assistance. One advantage of conducting the survey in person was that interviewers could answer any questions immediately. Some of the patients also needed assistance with the tablet PC used to record their responses.

The single inclusion criterion for physicians was professional activity in the field of gastroenterology. We decided to interview gastroenterologists, as they are responsible for liver cancer patients, usually present patient cases at the tumor board, and are experts on the various therapy options. We made the first contact with physicians via a personal letter. If we received no reply after 3 weeks, we sent a first reminder by e-mail, followed by a second reminder after another 3 weeks. We informed all participants about the study’s background and how we would store their personal data.

The inclusion criteria for medical laymen were age (> 18 years old) and the ability to participate in an online survey.

### Data measurement and statistical analysis

We only included participants who completed the MaxDiff and the demographics section in full in our analyses. We evaluated the MaxDiff data using count analysis and Hierarchical Bayes estimation (HB) in Sawtooth Software. The former counts the number of times participants selected an attribute as “best” or “worst”, following Li et al.^42^, resulting in a rank order over all attributes. We used an HB to calculate individual scores under the logit rule^43^, receiving an overall ranked and scaled score (i.e., a part-worth utility order) for each clinical attribute. We examined the significance of each part-worth utility order by checking whether the corresponding confidence intervals overlapped.

In addition, regarding the retrospective data, we examined whether there were group differences, for example, in whether the patient had previous experience with surgery or had received chemotherapy. We determined these group differences by conducting t-test analyses, with a p-value ≤ 0.05 indicating statistical significance. We measured the effect sizes (the size of the difference) of the group comparisons using Cohen’s d.

### Ethical approval

All procedures performed in studies involving human participants were in accordance with ethical standards of the institutional and/or national research committee and with the 1964 Helsinki declaration and its later amendments or comparable ethical standards. The ethics committee of the Hannover Medical School has reviewed and approved all data management and outcomes related activities of this research project (Reference number: 3561-2017).

### Informed consent

Informed consent was obtained from all individual participants included in the study.

## Results

### Participants

In total, we included 261 participants (patients, physicians, and medical laymen) in this study, meaning the number of participants was below the calculated sample size of 117 per group. However, when analyzing the preliminary results of the first interviews, we noticed a homogeneity among the answers that compensated for the overall smaller number of participants.

Patients: 97 eligible patients participated in the survey. One patient dropped out during participation; therefore, we used 96 questionnaires for the analysis.

Physicians: Over 24 months, we contacted 948 gastroenterologists from various hospitals in Germany via a personal letter. Upon completion of the survey period, we achieved a response rate of almost 9%, giving us 75 sets of data from physicians for evaluation. The physicians differed in terms of their career level (48% resident, 14.7% fellow physician, 28% attending physician, and 8% chief of service).

Medical laymen: We recruited medical laymen using hroot^44^ from a subject pool hosted by the Magdeburg Experimental Laboratory of Economic Research consisting mostly of students from various faculties of the University of Magdeburg. In total, we recruited 89 medical laymen.

Table 3 summarizes the study population’s descriptive data. All three groups gave information about their previous experience with surgery and whether they had previous experience with minimally invasive procedures.

**Table 3:** Description of the study population.

	Patients	Physicians	Medical laymen
Total, $N$	96	75	89
**Sex, **${\varvec{n}}$** (%)**			
Women	36 (37.5)	28 (37.3)	41 (46.1)
Men	60 (62.5)	47 (62.7)	48 (53.9)
**Age (years)**			
Mean	66	36	25
Standard deviation	11.38	8.59	3.27
**Experiences with surgery (Wording of the question: “Have you ever had surgery?”.), **${\varvec{n}}$** (%)**			
Yes	90 (93.8)	44 (59.5)	42 (47.2)
No	6 (6.2)	30 (40.5)	47 (52.8)
**Experiences with minimally invasive procedures (Wording of the question: “Have you ever had a minimally invasive procedure?”.), **${\varvec{n}}$** (%)**			
Yes	68 (70.8)	23 (31.1)	34 (38.2)
No	28 (29.2)	51 (68.9)	55 (61.8)

### Maximum difference scaling—results

We (1) received a rank order over all clinical attributes (count analysis) and (2) determined the individual part-worth utilities (HB) for each participant. Table 4 presents the best and worst count proportion and the absolute MaxDiff attribute ranking. We asked participants to classify clinical attributes as either the most or least important. The rankings are based on the best–worst percentage differences. Although the rank orders of patients and physicians differed only in the three least important attributes, medical laymen ranked the clinical attributes differently. However, all three groups rated the first four attributes as the most important.

**Table 4:** Absolute MaxDiff clinical attributes ranking – count analysis.

Attribute no.	Attribute	Best and (worst) count proportion/rankorder of attributes							
		Aggregated data Best (Worst)^a^	Rank order aggregated	Patients Best (Worst)^a^	Rank order patients	Physicians Best (Worst)^a^	Rank order physicians	Medical laymen Best (Worst)^a^	Rank order medical laymen
1	Probability (certainty) of a complete removal of the tumor	0.808 (0.023)	1	0.812 (0.034)	1	0.800 (0.019)	1	0.812 (0.016)	1
2	Probability of reoccurrence of the disease	0.750 (0.030)	2	0.694 (0.051)	2	0.794 (0.025)	2	0.772 (0.013)	2
3	Pathological evidence of tumor removal	0.651 (0.072)	3	0.650 (0.087)	3	0.721 (0.046)	3	0.593 (0.079)	4
4	Possible complications during the medical intervention	0.605 (0.060)	4	0.521 (0.104)	4	0.671 (0.032)	4	0.642 (0.034)	3
5	Welfare after the medical intervention	0.402 (0.166)	5	0.415 (0.147)	5	0.411 (0.129)	5	0.381 (0.218)	7
6	Duration and intensity of the pain	0.345 (0.196)	6	0.323 (0.207)	6	0.331 (0.192)	6	0.381 (0.189)	6
7	Degree of difficulty of the medical intervention	0.340 (0.198)	7	0.311 (0.212)	7	0.318 (0.225)	7	0.391 (0.161)	5
8	Frequency of follow-up care	0.235 (0.345)	8	0.277 (0.340)	8	0.140 (0.436)	8	0.268 (0.273)	8
9	Type of anesthesia	0.165 (0.449)	9	0.228 (0.379)	9	0.106 (0.497)	9	0.147 (0.484)	9
10	Duration of hospitalization	0.114 (0.547)	10	0.173 (0.493)	10	0.102 (0.525)	10	0.061 (0.625)	12
11	Length of anesthesia	0.089 (0.532)	11	0.125 (0.462)	11	0.090 (0.534)	11	0.050 (0.605)	11
12	Cost of therapy	0.075 (0.612)	12	0.039 (0.751)	14	0.084 (0.573)	12	0.105 (0.496)	10
13	Size of the scar	0.046 (0.690)	13	0.036 (0.689)	13	0.056 (0.758)	14	0.050 (0.634)	13
14	Profit of the hospital as a result of the treatment choice	0.037 (0.748)	14	0.060 (0.710)	12	0.035 (0.684)	13	0.013 (0.841)	14

^a^The first value indicates the Best Count proportion, values given in the parenthesis indicates the Worst Count proportion.

Because this ranking only shows the order of the clinical attributes for each group, from the most to the least important, we can draw no conclusions about the individual clinical attributes’ part-worth utility. Table 5 presents the results of the corresponding hierarchical Bayes analyses, allowing the part-worth utility of each of the 14 clinical attributes and the associated confidence intervals to be presented.

**Table 5:** Part-worth utility of each clinical attribute – hierarchical Bayes analyses.

Attribute no.	Attribute	Patients			Physicians			Medical Laymen		
		Av	95% lower	95% upper	Av	95% lower	95% upper	Av	95% lower	95% upper
1	Probability (certainty) of a complete removal of the tumor	15.829	15.457	16.201	16.471	16.224	16.718	15.694	15.495	15.892
2	Probability of reoccurrence of the disease	15.017	14.533	15.500	16.521	16.285	16.758	15.725	15.520	15.931
3	Pathological evidence of tumor removal	14.056	13.392	14.719	16.063	15.732	16.393	13.913	13.307	14.518
4	Possible complications during the medical intervention	12.438	11.835	13.041	16.291	16.041	16.541	14.852	14.519	15.186
5	Welfare after the medical intervention	10.172	9.457	10.887	11.121	10.123	12.119	8.363	7.356	9.369
6	Duration and intensity of the pain	8.491	7.656	9.325	9.227	8.218	10.236	9.641	9.004	10.278
7	Degree of difficulty of the medical intervention	7.802	7.037	8.568	7.744	6.785	8.703	10.708	9.857	11.558
8	Frequency of follow-up care	5.185	4.404	5.965	1.672	1.233	2.110	5.539	4.776	6.302
9	Type of anesthesia	4.260	3.479	5.042	0.892	0.680	1.103	2.175	1.480	2.869
10	Duration of hospitalization	2.505	1.939	3.070	0.913	0.616	1.209	0.457	0.332	0.583
11	Length of anesthesia	2.335	1.858	2.811	0.816	0.537	1.095	0.445	0.324	0.566
12	Cost of therapy	0.430	0.279	0.582	1.006	0.563	1.449	1.869	1.247	2.491
13	Size of the scar	0.446	0.332	0.560	0.568	0.096	1.040	0.380	0.267	0.493
14	Profit of the hospital as a result of the treatment choice	1.036	0.530	1.542	0.696	0.308	1.085	0.239	0.046	0.433

We can rank the attributes in a clear order and derive relationships between attributes using probability scale values of the HB^45^. That means, for example, the attribute of probability of complete removal of the tumor (utility of 15) is about twice as important to patients as the attribute of degree of difficulty of medical intervention (utility of 7). Nevertheless, it should be noted that the part-worth utilities are subject to model-based uncertainty, as their representation as 95% confidence intervals shows. If the confidence intervals of two neighboring attributes do not overlap, we can be at least 95% confident that the participants preferred the higher-ranked attribute over the other^46^. When the confidence intervals do overlap, the confidence decreases accordingly.

Based on the estimated part-worth utilities (see Table 5), we see that all three groups rated the attributes A1 (probability (certainty) of a complete removal of the tumor) and A2 (probability of reoccurrence of the disease) as the most important. A3 (pathological evidence of tumor removal) and A4 (possible complications during the medical intervention) follow the first two attributes at a slight distance.

**Figure 2:**
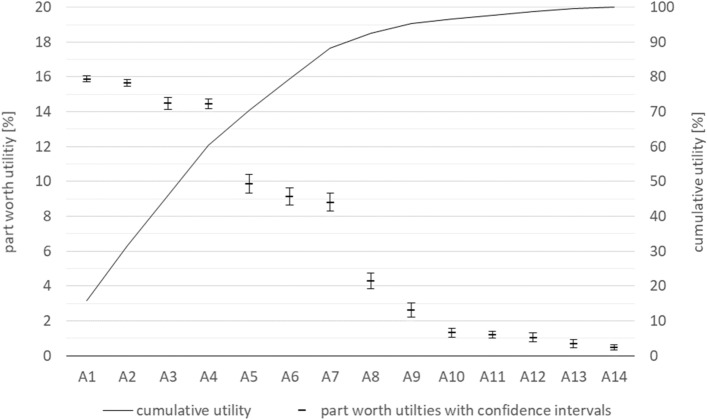
Joint order of attributes and part-worth utilities with confidence intervals and cumulative utility.

It becomes clear that the three groups assessed the attributes similarly—in particular, all groups assessed attributes A1 to A7 as the most important—and that these attributes provided similar part-worth utilities across all groups. Based on these similarities, we reanalyzed the pooled data to derive a joint order of all attributes (see Fig. 2). We found that for all groups, the cumulative part-worth utilities of attributes A1 to A7 amounted to approximately 90% of the total utility. The remaining 10% of the total utility was divided among attributes A8 to A14.

### Explorative findings

To investigate whether the patients’ age affected their evaluation of the attributes, we first performed a median split and divided the patients into two subgroups: (1) younger than 67 years and (2) older than or equal to 67 years. When comparing the determined probability scale values for both subgroups, we found that attribute A4 (possible complications during medical intervention) is significantly more important (t(76.719) = -1.953, p ≤ 0.05) for patients in subgroup 1 (M = 13.023; SD = 2.24; n = 49), than it is for patients in subgroup 2 (M = 11.828; SD = 3.577; n = 47). Cohen’s d = 0.401, so we consider this a medium-size effect.

We performed further evaluations and found, for example, that for patients older than 67, attributes A10 (duration of hospitalization) and A11 (length of anesthesia) were significantly more important than for patients younger than 67. However, we have not analyzed these results in more detail, as the part-worth utility of these attributes is below 2%, and therefore, we do not consider them relevant.

The other comparisons (e.g., based on the current ECOG performance status or whether the patient had previously been treated for cancer) revealed no relevant group differences.

## Discussion

We aimed to identify patients’, physicians’, and medical laymen’s preferences regarding decision-making in liver cancer therapy. We designed the study as a two-phase study. In this first part, we narrowed down 14 clinical attributes (identified through a literature review) to the most relevant ones using the MaxDiff scaling methodology. We found that all three groups rated the same four clinical attributes (probability (certainty) of a complete removal of the tumor, probability of reoccurrence of the disease, pathological evidence of tumor removal, and possible complications during the medical intervention) as the most important. Apparently, all three groups perceived these four clinical attributes as causally related to the success and quality of therapy.

Basically, our results regarding relevant attributes for liver cancer therapies align with previous findings that the attributes concerning overall survival are most important (e.g.,^2^), followed by attributes concerning risk of side effects and adverse events^2^. Additionally, we found that attributes regarding treatment time, comfortability, and administration are also considered relevant, which supports findings from Chiba et al.^40^, Lo et al.^2^, and Musa et al.^35^. However, we found that attributes related to costs of therapies, which Musa et al.^35^ considered relevant, were less important.

As a result of their study, Molinari et al.^27^ suggested that “patients’ values and attitudes toward risks and benefits for the treatment of ES-HCC should be explicitly elicited and included in multidisciplinary treatment decisions.” This result seems particularly relevant in light of Chen et al.^47^ finding of “a difference between liver cancer patients’ treatment preferences and their physicians’ recommendations.” In contrast to the latter, we found that patients and physicians (and medical laymen) considered the same attributes relevant. The earlier, contradictory results by Chen et al.^47^ can possibly be explained by the design of tumor board decisions, which primarily concern oncological outcomes rather than the specific preferences of physicians and patients. In this regard, our study strongly recommends eliciting patients’ preferences explicitly, in line with Molinari et al.^27^, and implementing these preferences in the decision-making process of interdisciplinary tumor boards. This would support the idea of shared decision-making by putting the patient even more at the center of the treatment decision. This is particularly important, as in the absence of a preference assessment for patients, physicians may replace their patients’ preferences with their own preferences^48–50^. Knowing that the average preferences of patients and their treating physicians are not far apart might defuse this conflict and further suggests that a patient can be somewhat confident that their treating physician’s professional perception is in line with the patient’s interests. However, our results cannot answer the question of whether both groups would arrive at the same choice of therapy, which offers a starting point for further research.

On the other hand, physicians often recommend the treatment in which they specialize. For example, surgeons are more likely to recommend surgery than non-surgeons. In recognition of this, some physicians inform their patients about their specialty bias^51^. This disclosure decreases the information gap between the physician and their patient and, theoretically, allows the patient to make a more informed decision^52^. Yet, practically, patients might either ignore the information or even lose trust in their advisors^51–53^. Knowing the relevant attributes of therapy choices for the average patient might be helpful as a directive for physicians or tumor-board participants who are aware of their biases.

Knowledge of a cancer-patient cohort’s central therapy preferences might be valuable for the individual patients and physician. Cancer patients are often confronted with an overwhelming amount of diagnoses, information, and decisions, which can be emotionally draining and distracting. It might be helpful to know how other patients weight benefits and risks of treatment options. This would allow physicians to guide their patients through the decision-making process based on what most patients suffering from the same disease would rate as the most important attributes.

Interestingly, the rank order of patients and physicians differed only in the three least important attributes, whereas medical laymen ranked the attributes differently. It remains unclear why medical laymen ranked “welfare after the medical intervention” and “duration of hospitalization” as less important and considered “cost of therapy” and “degree of difficulty of the medical intervention” more important. A possible explanation could be the homogeneity of the medical laymen group. They were recruited from a pool of mostly students who were younger and, presumably, had better health status compared to the other groups.

Further limitations need to be considered. The study group was somewhat inhomogeneous, which hampers generalizability and limits our statistical analysis. First, this was a national study, and second, it was performed on a sample of German gastroenterologists and on a sample of German patients, which may limit the generalizability of our results. Within this limitation, we managed to acquire a heterogeneous study group with physicians on various career levels. Third, the laymen were mostly students with a significant age difference to the patient group.

Furthermore, we are aware of an additional (potential) limitation related to the directionality of the used MaxDiff attributes. In this regard, one could argue that some attributes may not have clear directionality. However, we took great care to formulate all attributes in such a way that the direction should be clear to participants, in particular, that all participants interpret the direction of the attributes in a similar way.

Evaluating relevant clinical attributes regarding the choice of liver cancer therapy and identifying the most important among these were necessary, as the upcoming second phase of our IRALCT study is intended to further analyze the assessed clinical attributes using a choice-based conjoint (CBC) analysis. For the CBC analysis, the number of attributes should not exceed nine to ensure a feasible number of subjects and avoid cognitively overloading the participants. However, in the second study phase, we will expand our approach to determine and include relevant behavioral and social attributes.

## Conclusion

Our results show that the preferences of the physicians, patients, and medical laymen were very similar and stable. Our study provides valuable information that can support shared decision-making by highlighting which attributes may require further attention.

## Supplementary Information

Supplementary Information.
