# Supplementary material for: Identification of Relevant Attributes for Liver Cancer Therapies (IRALCT): a maximum-difference-scaling analysis
Source: Sci Rep. 2022 Nov 9;12:19143. doi: 10.1038/s41598-022-23097-w (PMC9646805; doi:10.1038/s41598-022-23097-w)
Supplement: Supplementary file 1 — Supplementary Information. [file 41598_2022_23097_MOESM1_ESM.docx]

**Identification of Relevant Attributes for Liver Cancer Therapies (IRALCT): A Maximum-Difference-Scaling Analysis**

(Bennet Hensen, Carolin Winkelmann, Frank K. Wacker, Bodo Vogt,
Cornelia L. A. Dewald, Thomas Neumann)

**Appendix**

In this appendix, we aim to describe the *medical* value of the attributes chosen for our study.

A1 Probability (certainty) of a complete removal of the tumor: For minimally invasive therapy to be successful, the tumor burden must be reduced, or the tumor must go into remission. Therefore, many clinical studies have assessed the probability of tumor removal under a therapy, e.g., a surgical resection, minimal invasive therapies such as SIRT and TACE [1], chemosaturation [2], and liver ablation.

A2 Probability of reoccurrence of the disease: Knowing the probability of recurrence of a disease is essential for providing information about a patient’s likelihood of developing the disease again in the future, depending on the chosen therapy [3–6].

A3 Pathological evidence of tumor removal: The pathological evidence of complete tumor removal can only be obtained following surgical removal of a tumor. The purpose of this report is to provide information about the size, location, and type of tumor that was removed, as well as the extent of tumor invasion and any lymph node involvement. Additionally, the pathology report will provide details about the surgical margins (the distance from the tumor to the edge of the surgical incision) as well as whether cancer cells were found at the edge of the surgical incision. Surgical removal should involve resection of the cancer lesion with a large enough margin to prevent recurrence to best ensure prolonged survival [7]. This attribute was selected to investigate whether patients rate the pathologically proven removal of their tumor as important, or whether indirect evidence such as an absence of contrast uptake indicating necrosis on imaging as a sign of tumor removal might be sufficient [8].

A4 Possible complications during the medical intervention: To give fully informed consent for a medical intervention, patients need to understand the risks and benefits of a proposed treatment. To make an informed decision regarding whether to undergo a medical intervention, patients must know what complications may arise, as complication rates differ between treatments. For example, a minimally invasive ablation involves less severe adverse events [9, 10] than an open resection [8, 11, 12].

A5 Welfare after the medical intervention: This attribute describes the patient’s state of health after treatment, including physical discomfort, pain, and ability to carry out daily activities [13].

A6 Duration and intensity of the pain: Anxiety and distress are caused by the fear of pain during and after procedures [14]. It is imperative that pain and anxiety are appropriately managed to perform medical procedures safely and successfully [15].

A7 Degree of difficulty of the medical intervention: The complexity of a medical intervention can affect the length of time required for the intervention and the level of training needed by the treating physician. As complicated medical interventions are often high-risk and require close monitoring by specialists, they should be performed in specialized medical centers, where patients are provided with the best care possible. In addition, these centers often have the most advanced technology and equipment available, which is beneficial for the success of the procedure [16].

A8 Frequency of follow-up care: The frequency of follow-up care can impact how often the patient needs to return to the hospital for treatment and checkups. Routine follow-ups after cancer treatment are important for providing supportive care and monitoring treatment effects [17].

A9 Type of anesthesia: The type of anesthesia used can affect how long the patient is sedated and how much pain they experience [18]. Anesthesia techniques also have a role in postoperative complications and length of stay [19]. Regional anesthesia could improve long-term mortality, cancer recurrence, cognitive dysfunction, persistent chronic pain, and rehabilitation in the postoperative period [20].

A10 Duration of hospitalization: The duration of hospitalization can impact how long the patient needs to stay in the hospital overall, which could influence patient satisfaction [21, 22].

A11 Length of anesthesia: The length of anesthesia can affect how long the patient is sedated and how comfortable they feel following the procedure. Additionally, prolonged anesthesia duration is associated with increased odds of complications [23].

A12 Cost of therapy: The cost of therapy can impact overall health care spending [24].

A13 Size of the scar: The size of a scar may affect the patient’s self-image following treatment. The appearance of surgical scars can affect patients’ social interactions and psychosocial health. Physicians and patients should agree on the ideal characteristics of a surgical scar to ensure that expectations are aligned [25, 26].

A14 Profit of the hospital as a result of the treatment choice: The profit for a hospital as a result of a treatment choice can impact how affordable treatment is for the medical system [24].

**References**

1. Vilgrain V, Pereira H, Assenat E, Guiu B, Ilonca AD, Pageaux G-P, et al. Efficacy and safety of selective internal radiotherapy with yttrium-90 resin microspheres compared with sorafenib in locally advanced and inoperable hepatocellular carcinoma (SARAH): an open-label randomised controlled phase 3 trial. The Lancet Oncology. 2017;18:1624–36. doi:10.1016/S1470-2045(17)30683-6.

2. Hughes MS, Zager J, Faries M, Alexander HR, Royal RE, Wood B, et al. Results of a Randomized Controlled Multicenter Phase III Trial of Percutaneous Hepatic Perfusion Compared with Best Available Care for Patients with Melanoma Liver Metastases. Ann Surg Oncol. 2016;23:1309–19. doi:10.1245/s10434-015-4968-3.

3. Abdalla EK, Vauthey J-N, Ellis LM, Ellis V, Pollock R, Broglio KR, et al. Recurrence and outcomes following hepatic resection, radiofrequency ablation, and combined resection/ablation for colorectal liver metastases. Ann Surg. 2004;239:818-25; discussion 825-7. doi:10.1097/01.sla.0000128305.90650.71.

4. Clasen S, Rempp H, Hoffmann R, Graf H, Pereira PL, Claussen CD. Image-guided radiofrequency ablation of hepatocellular carcinoma (HCC): is MR guidance more effective than CT guidance? Eur J Radiol. 2014;83:111–6. doi:10.1016/j.ejrad.2013.09.018.

5. Cosimelli M, Golfieri R, Cagol PP, Carpanese L, Sciuto R, Maini CL, et al. Multi-centre phase II clinical trial of yttrium-90 resin microspheres alone in unresectable, chemotherapy refractory colorectal liver metastases. Br J Cancer. 2010;103:324–31. doi:10.1038/sj.bjc.6605770.

6. Lammer J, Malagari K, Vogl T, Pilleul F, Denys A, Watkinson A, et al. Prospective randomized study of doxorubicin-eluting-bead embolization in the treatment of hepatocellular carcinoma: results of the PRECISION V study. Cardiovasc Intervent Radiol. 2010;33:41–52. doi:10.1007/s00270-009-9711-7.

7. Lafaro K, Grandhi MS, Herman JM, Pawlik TM. The importance of surgical margins in primary malignancies of the liver. J Surg Oncol. 2016;113:296–303. doi:10.1002/jso.24123.

8. Galle PR, Forner A, Llovet JM, Mazzaferro V, Piscaglia F, Raoul JL. EASL Clinical Practice Guidelines: Management of hepatocellular carcinoma. J Hepatol. 2018;69:182–236. doi:10.1016/j.jhep.2018.03.019.

9. Shiina S, Tateishi R, Arano T, Uchino K, Enooku K, Nakagawa H, et al. Radiofrequency ablation for hepatocellular carcinoma: 10-year outcome and prognostic factors. Am J Gastroenterol. 2012;107:569-77; quiz 578. doi:10.1038/ajg.2011.425.

10. Solbiati L, Ahmed M, Cova L, Ierace T, Brioschi M, Goldberg SN. Small liver colorectal metastases treated with percutaneous radiofrequency ablation: local response rate and long-term survival with up to 10-year follow-up. Radiology. 2012;265:958–68. doi:10.1148/radiol.12111851.

11. Cheung TT, Poon RTP, Yuen WK, Chok KSH, Jenkins CR, Chan SC, et al. Long-term survival analysis of pure laparoscopic versus open hepatectomy for hepatocellular carcinoma in patients with cirrhosis: a single-center experience. Ann Surg. 2013;257:506–11. doi:10.1097/SLA.0b013e31827b947a.

12. Haas RJ de, Wicherts DA, Flores E, Azoulay D, Castaing D, Adam R. R1 resection by necessity for colorectal liver metastases: is it still a contraindication to surgery? Ann Surg. 2008;248:626–37. doi:10.1097/SLA.0b013e31818a07f1.

13. Fan S-Y, Eiser C, Ho M-C. Health-related quality of life in patients with hepatocellular carcinoma: a systematic review. Clin Gastroenterol Hepatol. 2010;8:559-64.e1-10. doi:10.1016/j.cgh.2010.03.008.

14. Iyer VR, Sheedy SP, Gunderson TM, Christopherson ML, Kurup AN, Schmitz JJ, Atwell TD. Procedure-Related Pain During Image-Guided Percutaneous Biopsies: A Retrospective Study of Prevalence and Predictive Factors. AJR Am J Roentgenol. 2019;213:755–61. doi:10.2214/AJR.19.21248.

15. Martin ML, Lennox PH. Sedation and analgesia in the interventional radiology department. J Vasc Interv Radiol. 2003;14:1119–28. doi:10.1097/01.rvi.0000086536.86489.82.

16. Aust JB, Henderson W, Khuri S, Page CP. The impact of operative complexity on patient risk factors. Ann Surg. 2005;241:1024-7; discussion 1027-8. doi:10.1097/01.sla.0000165196.32207.dd.

17. Rose PW, Watson E. What is the value of routine follow-up after diagnosis and treatment of cancer? Br J Gen Pract. 2009;59:482–3. doi:10.3399/bjgp09X453512.

18. Kehlet H, Dahl JB. Anaesthesia, surgery, and challenges in postoperative recovery. The Lancet. 2003;362:1921–8. doi:10.1016/S0140-6736(03)14966-5.

19. Oh TT, Martel CG, Clark AG, Russo MB, Nossaman BD. Impact of Anesthetic Predictors on Postpartum Hospital Length of Stay and Adverse Events Following Cesarean Delivery: A Retrospective Study in 840 Consecutive Parturients. Ochsner J. 2015;15:228–36.

20. Lois F, Kock M de. Does regional anesthesia improve long-term patient outcome? Techniques in Regional Anesthesia and Pain Management. 2008;12:203–8. doi:10.1053/j.trap.2008.09.006.

21. Diwan W, Nakonezny PA, Wells J. The Effect of Length of Hospital Stay and Patient Factors on Patient Satisfaction in an Academic Hospital. Orthopedics. 2020;43:373–9. doi:10.3928/01477447-20200910-02.

22. Tsai TC, Orav EJ, Jha AK. Patient satisfaction and quality of surgical care in US hospitals. Ann Surg. 2015;261:2–8. doi:10.1097/SLA.0000000000000765.

23. Phan K, Kim JS, Kim JH, Somani S, Di’Capua J, Dowdell JE, Cho SK. Anesthesia Duration as an Independent Risk Factor for Early Postoperative Complications in Adults Undergoing Elective ACDF. Global Spine J. 2017;7:727–34. doi:10.1177/2192568217701105.

24. Musa MS, Ozsahin DU, Ozsahin I. A Comparison for Liver Cancer Treatment Alternatives. In: Musa MS, Ozsahin DU, Ozsahin I, editors. 2019 Advances in Science and Engineering Technology International Conferences (ASET); 26.03.2019 - 10.04.2019; Dubai, United Arab Emirates: IEEE; 2019. p. 1–4. doi:10.1109/ICASET.2019.8714471.

25. Young VL, Hutchison J. Insights into patient and clinician concerns about scar appearance: semiquantitative structured surveys. Plast Reconstr Surg. 2009;124:256–65. doi:10.1097/PRS.0b013e3181a80747.

26. Zhang J, Miller CJ, O’Malley V, Bowman EB, Etzkorn JR, Shin TM, Sobanko JF. Patient and Physician Assessment of Surgical Scars: A Systematic Review. JAMA Facial Plast Surg. 2018;20:314–23. doi:10.1001/jamafacial.2017.2314.
